# *YTHDC1* gene polymorphisms and neuroblastoma susceptibility in Chinese children

**DOI:** 10.18632/aging.203760

**Published:** 2021-12-12

**Authors:** Yong Li, Tongyi Lu, Jian Wang, Zhenjian Zhuo, Lei Miao, Zhonghua Yang, Jiao Zhang, Jiwen Cheng, Haixia Zhou, Suhong Li, Li Li, Jing He, Aiwu Li

**Affiliations:** 1 Department of Pediatric Surgery, Qilu Hospital, Cheeloo College of Medicine, Shandong University, Jinan 250012, Shandong, China; 2 Department of Pediatric Surgery, Hunan Children’s Hospital, Changsha 410004, Hunan, China; 3 Department of Pediatric Surgery, Guangzhou Institute of Pediatrics, Guangdong Provincial Key Laboratory of Research in Structural Birth Defect Disease, Guangzhou Women and Children’s Medical Center, Guangzhou Medical University, Guangzhou 510623, Guangdong, China; 4 Department of Pediatric Surgery, Shengjing Hospital of China Medical University, Shenyang 110004, Liaoning, China; 5 Department of Pediatric Surgery, The First Affiliated Hospital of Zhengzhou University, Zhengzhou 450052, Henan, China; 6 Department of Pediatric Surgery, The Second Affiliated Hospital of Xi’an Jiaotong University, Xi’an 710004, Shaanxi, China; 7 Department of Hematology, The Second Affiliated Hospital and Yuying Children’s Hospital of Wenzhou Medical University, Wenzhou 325027, Zhejiang, China; 8 Department of Pathology, Children Hospital and Women Health Center of Shanxi, Taiyuan 030013, Shannxi, China; 9 Kunming Key Laboratory of Children Infection and Immunity, Yunnan Key Laboratory of Children’s Major Disease Research, Yunnan Institute of Pediatrics Research, Yunnan Medical Center for Pediatric Diseases, Kunming Children’s Hospital, Kunming 650228, Yunnan, China

**Keywords:** neuroblastoma, m^6^A, *YTHDC1*, polymorphism, susceptibility

## Abstract

Neuroblastoma (NB) is the most common extracranial tumor in children. YTHDC1, a member of RNA methylation modification binding proteins, plays critical roles in tumor occurrence and metastasis. However, it is unclear whether *YTHDC1* gene polymorphisms are related to NB susceptibility. Herein, we aimed to evaluate the association between *YTHDC1* gene polymorphisms (rs2293596 T>C, rs2293595 T>C, rs3813832 T>C) and susceptibility of NB by logistic regression models. In this eight-center case-control study, 898 patients with NB and 1734 healthy controls were genotyped by TaqMan assay. The results showed that rs3813832 TC genotype could significantly reduce the susceptibility of NB compared with the TT genotype [adjusted odds ratio (AOR) = 0.81, 95% confidence interval (CI) = 0.68–0.96, *P* = 0.018]. Combined genotype analysis revealed that individuals with 3 protective genotypes had a prominently lower NB risk than those with 0-2 protective genotypes (AOR = 0.80, 95% CI = 0.68–0.94, *P* = 0.006). The stratified analysis also demonstrated the protective effect of rs3813832 TC/CC and 3 protective genotypes in certain subgroups. Further functional experiments revealed that *YTHDC1* siRNA-554, targeting the area near the rs3813832 T>C polymorphism site, could observably inhibit the proliferation and migration of NB cells. In conclusion, our findings highlight the involvement of *YTHDC1* gene and its genetic variants in the etiology of NB.

## INTRODUCTION

Neuroblastoma (NB) is the most common extracranial solid tumor in children, accounting for 8% of all pediatric malignancies [1]. The mortality rate of NB is 0.85–1.1 cases per 100,000 children in the world [2]. Most of the low-risk NB patients resolve spontaneously without chemotherapy [3]. However, high-risk patients, accounting for nearly 50% of NB, have been extensively metastatic at the time of diagnosis. Despite various treatments such as surgery, chemotherapy, radiotherapy, and immunotherapy, the survival rate is still less than 40% [4]. Unfortunately, the pathogenesis of NB has not yet been fully understood [5, 6]. Studies have found that about 1–2% of NB cases are familial [2]. *PHOX2B* [7] or *ALK* [8] gene mutations are considered to be the main cause of familial NB. However, sporadic neuroblastoma is the main form of NB [9]. Increasing evidence shows that genetic variants play a key role in the development of NB [10–12]. In recent years, several NB susceptibility loci have been identified through genome-wide association studies (GWASs), such as *BARD1* [13], *LMO1* [14], *HACE1* [15], *TP53* [16], *CASC15* [17], *MLF1* [18], and *CDKN1B* [19]. Moreover, many polymorphisms were also identified to influence chemotherapy and outcome in patients with neuroblastoma [20, 21]. The identification of these susceptible loci has deepened the understanding of the pathogenesis of NB [22, 23]. However, more gene single nucleotide polymorphisms (SNPs) involved in the process of tumorigenesis await to be revealed.

N6-methyladenosine (m^6^A) is the most common and abundant post-transcriptional modification of eukaryotic mRNA [24, 25]. The formation of m^6^A is dynamically converted by methyltransferase complexes (“writers”; *METTL3*, *METTL14*, and *WTAP*), demethylases (“erasers”; *FTO* and *ALKBH5*), and binding proteins (“readers”; *YTHDF1/2/3*, *YTHDC1*) [26, 27]. Recently, it has been confirmed that m^6^A modified disorders are involved in human carcinogenesis, including osteosarcoma [28], glioblastoma [29], colorectal cancer [30], acute myeloid leukemia [31], gastric cancer [32], glioma [33], and bladder cancer [34]. The abnormal modification level of m^6^A may affect the individual’s cancer susceptibility [35]. YTH domain contains protein 1 (YTHDC1), an important m^6^A recognition protein, is involved in mRNA splicing and the export of methylated mRNAs [36, 37], but its role has not yet been deeply understood.

Considering the evidence that m^6^A is closely related to tumorigenesis and metastasis, we speculate that there may be a significant correlation between the genetic variation of m^6^A modified gene *YTHDC1* and the risk of NB. Therefore, we performed this study by recruiting 898 NB patients and 1734 controls to explore the relationship between *YTHDC1* SNPs and NB risk in Chinese children.

## RESULTS

### Association between *YTHDC1* gene polymorphisms and NB susceptibility

In this study, 896 NB cases and 1733 controls were successfully genotyped. The three polymorphisms of *YTHDC1* were consistent with Hardy-Weinberg equilibrium (HWE) (*P* = 0.696 for rs2293596 T>C, *P* = 0.556 for rs2293595 T>C, *P* = 0.968 for rs3813832 T>C). The genotype distribution frequencies of case and control groups were shown in Table 1. The results showed that in the rs3813832 T>C polymorphism, relative to the TT genotype, TC genotype could significantly reduce the susceptibility of NB [adjusted odds ratio (AOR) = 0.81, 95% confidence interval (CI) = 0.68–0.96, *P* = 0.018]. In addition, we used rs2293596 TT/TC, rs2293595 TC/CC, or rs3813832 TC/CC as protective genotypes to further investigate the combined effect of these three SNPs. The combined genotype analysis revealed that individuals carrying 3 protective genotypes had a prominent lower risk of NB compared with individuals carrying 0–2 protective genotypes (AOR = 0.80, 95% CI = 0.68–0.94, *P* = 0.006).

**Table 1:** Association between *YTHDC1* gene polymorphisms and neuroblastoma risk.

**Genotype**	**Cases** **(*N* = 896)**	**Controls** **(*N* = 1733)**	***P* ^a^**	**Crude OR** **(95% CI)**	***P***	**Adjusted OR** **(95% CI)^b^**	***P* ^b^**
rs2293596 T>C (HWE = 0.696)							
TT	580 (64.73)	1144 (66.01)		1.00		1.00	
TC	272 (30.36)	525 (30.29)		1.02 (0.86–1.22)	0.811	1.03 (0.86–1.23)	0.778
CC	44 (4.91)	64 (3.69)		1.36 (0.91–2.02)	0.132	1.36 (0.91–2.02)	0.130
Additive			0.283	1.08 (0.94–1.25)	0.283	1.08 (0.94–1.25)	0.267
Dominant	316 (35.27)	589 (33.99)	0.513	1.06 (0.89–1.25)	0.511	1.06 (0.90–1.26)	0.486
Recessive	852 (95.09)	1669 (96.31)	0.136	1.35 (0.91–1.99)	0.137	1.35 (0.91–2.00)	0.137
rs2293595 T>C (HWE = 0.556)							
TT	343 (38.28)	601 (34.68)		1.00		1.00	
TC	411 (45.87)	829 (47.84)		0.87 (0.73–1.04)	0.121	0.86 (0.72–1.03)	0.102
CC	142 (15.85)	303 (17.48)		0.82 (0.65–1.04)	0.107	0.82 (0.65–1.04)	0.107
Additive			0.070	0.90 (0.80–1.01)	0.070	0.90 (0.80–1.01)	0.067
Dominant	553 (61.72)	1132 (65.32)	0.068	0.86 (0.72–1.01)	0.068	0.85 (0.72–1.01)	0.059
Recessive	754 (84.15)	1430 (82.52)	0.289	0.89 (0.72–1.11)	0.289	0.89 (0.72–1.11)	0.309
rs3813832 T>C (HWE = 0.968)							
TT	499 (55.69)	902 (52.05)		1.00		1.00	
TC	314 (35.04)	697 (40.22)		**0.81 (0.69–0.97)**	**0.020**	**0.81 (0.68–0.96)**	**0.018**
CC	83 (9.26)	134 (7.73)		1.12 (0.83–1.50)	0.452	1.13 (0.84–1.52)	0.403
Additive			0.424	0.95 (0.84–1.08)	0.424	0.95 (0.84–1.08)	0.446
Dominant	397 (44.31)	831 (47.95)	0.076	0.86 (0.73–1.02)	0.076	0.86 (0.73–1.02)	0.075
Recessive	813 (90.74)	1599 (92.27)	0.176	1.22 (0.92–1.62)	0.177	1.24 (0.93–1.65)	0.148
Combined effect of protective genotypes^c^							
0–2	428 (47.77)	735 (42.41)		1.00		1.00	
3	468 (52.23)	998 (57.59)	0.009	**0.81 (0.69–0.95)**	**0.008**	**0.80 (0.68–0.94)**	**0.006**

Abbreviations: OR: odds ratio; CI: confidence interval; HWE: Hardy-Weinberg equilibrium. ^a^*χ*^2^ test for genotype distributions between neuroblastoma patients and cancer-free controls. ^b^Adjusted for age and gender. ^c^Protective genotypes were rs2293596 TT/TC, rs2293595 TC/CC and rs968697 TC/CC.

### Stratification analysis

Subsequently, we conducted a stratified analysis based on age, gender, sites of origin, and clinical stage to further explore the relationship between *YTHDC1* gene SNPs (rs2293596 T>C, rs2293595 T>C and rs3813832 T>C) and the risk of NB (Table 2). In the subgroup of clinical stage III + IV, the rs3813832 TC/CC genotype carriers suffer from a relatively lower risk of NB (AOR = 0.77, 95% CI = 0.62–0.96, *P* = 0.022). In addition, individuals with 3 protective genotypes had an observably decreased risk of NB (AOR = 0.80, 95% CI = 0.65–0.98, *P* = 0.032) in subgroup of children over 18 months of age. As to the participants’ gender, we found that male carrying the 3 protective genotypes also showed a lower NB risk (AOR = 0.77, 95% CI = 0.62–0.96, *P* = 0.021). From the sites of origin, individuals with 3 protective genotypes were less likely to have NB that original from mediastinum (AOR = 0.67, 95% CI = 0.51–0.90, *P* = 0.007). In terms of clinical stage, individuals carrying 3 protective genotypes displayed a weak susceptibility at clinical stages I + II + 4s (AOR = 0.78, 95% CI = 0.64–0.96, *P* = 0.019) and III + IV (AOR = 0.77, 95% CI = 0.62–0.97, *P* = 0.023).

**Table 2:** Stratification analysis for association between *YTHDC1* gene genotypes and neuroblastoma susceptibility.

**Variables**	**rs2293595** **(case/control)**		**AOR** **(95% CI)^a^**	***P* ^a^**	**rs3813832** **(case/control)**		**AOR** **(95% CI)^a^**	***P* ^a^**	**Protective genotypes** **(case/control)**		**AOR** **(95% CI)^a^**	***P* ^a^**
	**TT**	**TC/CC**			**TT**	**TC/CC**			**0-2**	**3**		
Age, month												
≤18	146/265	198/448	0.80 (0.62–1.04)	0.098	197/378	147/335	0.84 (0.65–1.09)	0.188	177/327	167/386	0.80 (0.62–1.03)	0.085
>18	197/336	355/684	0.89 (0.71–1.10)	0.284	302/524	250/496	0.87 (0.71–1.08)	0.205	251/408	301/612	**0.80 (0.65–0.98)**	**0.032**
Gender												
Female	156/265	250/479	0.88 (0.69–1.13)	0.325	222/396	184/348	0.94 (0.74–1.20)	0.639	191/318	215/426	0.83 (0.65–1.06)	0.131
Male	187/336	303/653	0.83 (0.66–1.04)	0.102	277/506	213/483	0.80 (0.65–1.00)	0.05	237/417	253/572	**0.77 (0.62–0.96)**	**0.021**
Sites of origin												
Adrenal gland	97/601	151/1132	0.82 (0.62–1.07)	0.143	145/902	103/831	0.76 (0.58–1.00)	0.051	117/735	131/998	0.81 (0.62–1.06)	0.119
Retroperitoneal	110/601	208/1132	0.99 (0.77–1.27)	0.93	164/902	154/831	1.02 (0.80–1.29)	0.888	141/735	177/998	0.91 (0.71–1.16)	0.43
Mediastinum	85/601	128/1132	0.80 (0.60–1.08)	0.143	121/902	92/831	0.83 (0.62–1.11)	0.206	111/735	102/998	**0.67 (0.51–0.90)**	**0.007**
Others	46/601	59/1132	0.69 (0.46–1.03)	0.07	64/902	41/831	0.70 (0.47–1.05)	0.087	52/735	53/998	0.76 (0.51–1.13)	0.178
Clinical stage												
I + II + 4s	184/601	285/1132	0.83 (0.67–1.02)	0.08	256/902	213/831	0.91 (0.74–1.11)	0.346	228/735	241/998	**0.78 (0.64–0.96)**	**0.019**
III + IV	150/601	244/1132	0.85 (0.67–1.07)	0.157	230/902	164/831	**0.77 (0.62–0.96)**	**0.022**	189/735	205/998	**0.77 (0.62–0.97)**	**0.023**

Abbreviations: AOR: adjusted odds ratio; CI: confidence interval. ^a^Adjusted for age and gender, omitting the corresponding stratify factor.

### Knockdown of *YTHDC1* significantly inhibits proliferation of neuroblastoma cells

**Figure 1:**
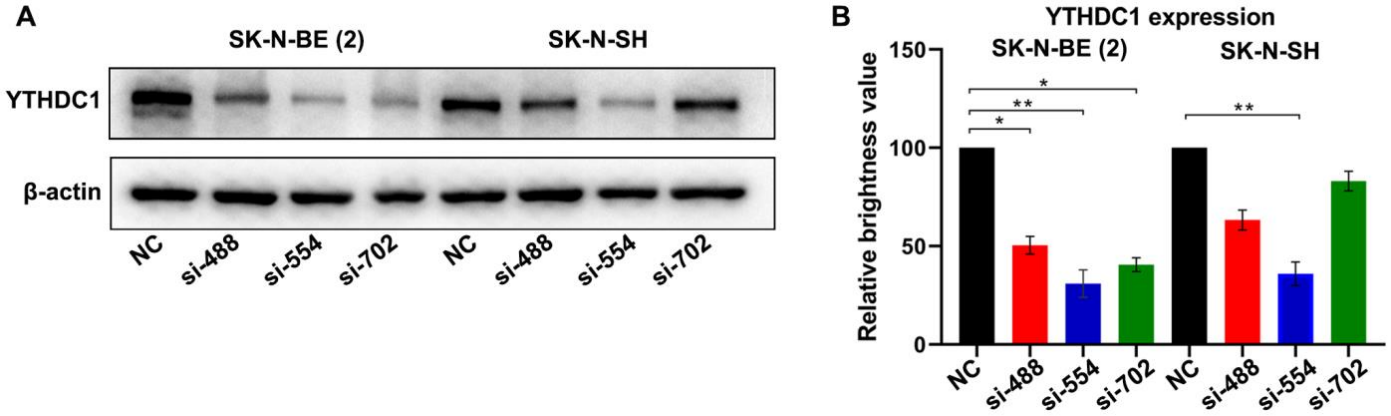
**Verification of the silencing effect of different interference sites of *YTHDC1* in NB cells.** (**A**) YTHDC1 expression in SK-N-BE (2) and SK-N-SH cells transfected with *YTHDC1* siRNAs (siRNA-488, siRNA-554, siRNA-702 and siRNA-NC) were detected by Western blot. (**B**) Quantitative analysis of YTHDC1 expression in SK-N-BE (2) and SK-N-SH cells.

SK-N-BE (2) and SK-N-SH cells transfected with *YTHDC1* siRNAs (siRNA-NC, siRNA-488, siRNA-554, and siRNA-702) were collected and lysed for Western blotting after 48 h (Figure 1A). The results showed that the expression level of YTHDC1 in SK-N-BE (2) cells transfected with siRNA-488/siRNA-554/siRNA-702 was about 50% (*P* < 0.05)/35% (*P* < 0.01)/42% (*P* < 0.05) compared with control group (Figure 1B). In SK-N-SH cells transfected with siRNA-488/siRNA-554/siRNA-702, the expression level *YTHDC1* was about 66% (*P* > 0.05)/39% (*P* < 0.01)/88% (*P* > 0.05). These results showed that *YTHDC1* siRNA could significantly reduce the expression of YTHDC1 in SK-N-BE (2) and SK-N-SH cells.

**Figure 2:**
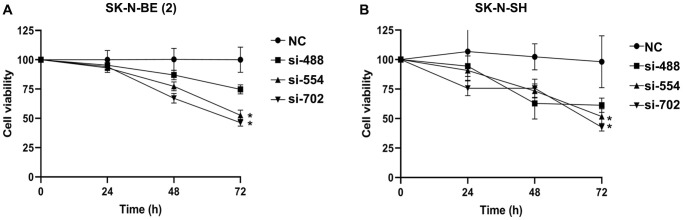
**The cell viability of NB transfected with *YTHDC1* siRNAs were measured by CCK-8 assay.** (**A**) The cell viability of SK-N-BE (2) transfected with *YTHDC1* siRNAs at 24 h, 48 h, 72 h. (**B**) The SK-N-SH cell viability transfected with *YTHDC1* siRNAs. Data were represented as the means ± SD. ^*^P < 0.05.

CCK-8 assay was used to evaluate the cell viability of SK-N-BE (2) and SK-N-SH transfected with *YTHDC1* siRNAs (Figure 2A, 2B). The results showed that the activities of SK-N-BE (2) and SK-N-SH cells transfected with siRNA-488 were not obviously affected in different time periods. The proliferation activities of SK-N-BE (2) and SK-N-SH cells transfected with siRNA-554 and siRNA-702 were suppressed dramatically at 72 h (*P* < 0.05). These results indicated that silencing *YTHDC1* would significantly inhibit the proliferation of NB cells.

### Knockdown of *YTHDC1* inhibits migration of neuroblastoma cells

**Figure 3:**
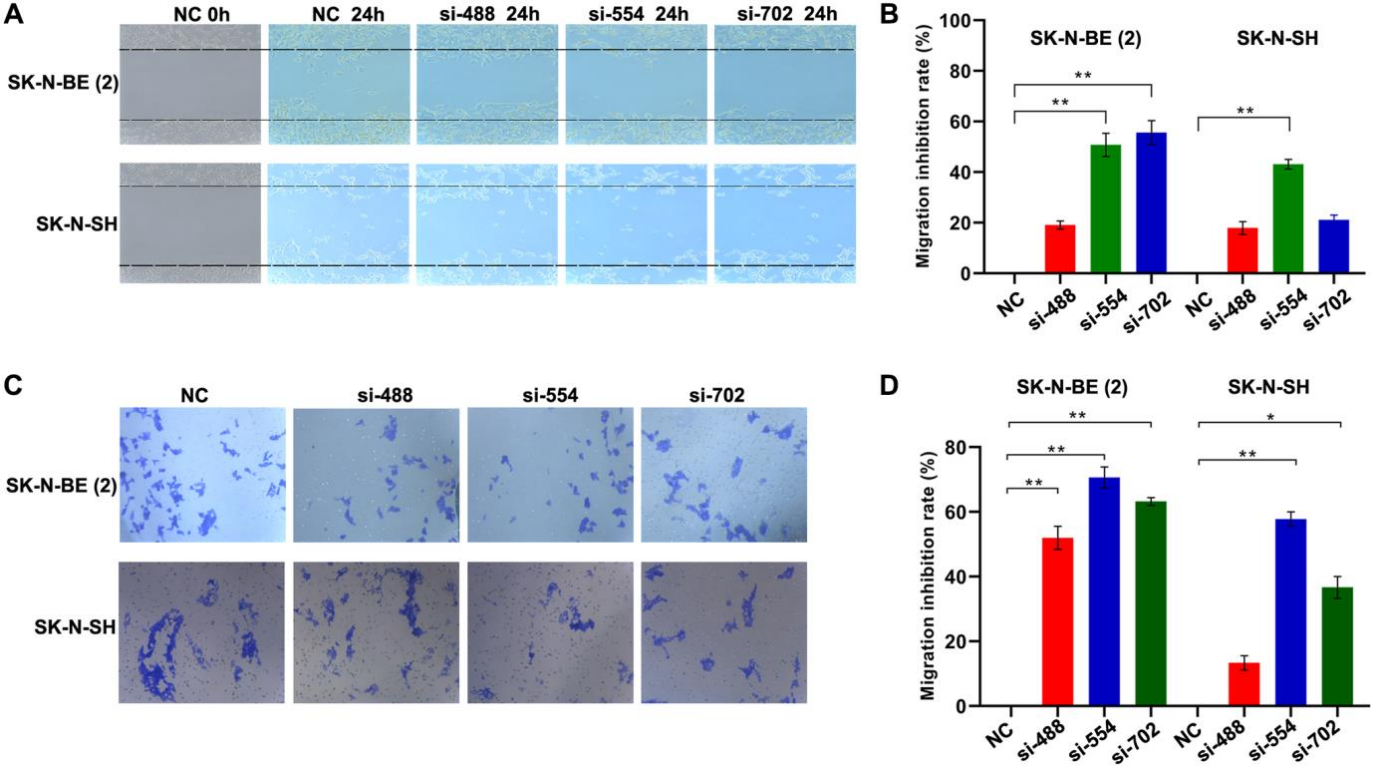
**Silencing *YTHDC1* inhibited NB cells migration.** (**A**) Migration ability of SK-N-BE (2) and SK-N-SH cells were evaluated by wound healing assay after transfection for 24 h. (**B**) Quantitative analysis of the migration inhibition rate of SK-N-BE (2) and SK-N-SH cells. (**C**) Identifying migration ability of SK-N-BE (2) and SK-N-SH cells transfected with *YTHDC1* siRNAs for 24 h by transwell migration assay. (**D**) Quantitative analysis of SK-N-BE (2) and SK-N-SH cell migration inhibition rate. Data were represented as the means ± SD, ^*^P < 0.05, ^**^P < 0.01.

We further investigated the effect of *YTHDC1* silencing on the migration of NB cells through wound healing assays (Figure 3A, 3B). These data showed that the migration inhibition rate of SK-N-BE (2) cells transfected with *YTHDC1* siRNA-488/554/702 reached 18% (*P* > 0.05)/50% (*P* < 0.01)/55% (*P* < 0.01). However, among SK-N-SH cells, only the cell line transfected with siRNA-554 was obviously suppressed in the migration ability, with an inhibition rate of 43%.

We also investigated the effect of *YTHDC1* silencing on the migration of NB cells through migration assays (Figure 3C, 3D). Migration assay results exhibited that the migration inhibition rate of SK-N-BE (2) cells transfected with siRNA-488/554/702 reached 51%/70%/63%, relative to the NC group (*P* < 0.01). The migration inhibition rate of SK-N-SH cells transfected with siRNA-488 was 13% (*P* > 0.05), whereas inhibition rate of cells transfected with siRNA-554 and siRNA-702 exceeded 57% and 40% (*P* < 0.01). These results indicate that silencing the expression of *YTHDC1* reduces the migration ability of the NB cells.

## DISCUSSION

In this study, we systematically evaluated the associations between common genetic variants in *YTHDC1* gene and NB risk. We further characterized the *YTHDC1* functionality in NB via molecular biology experiments. Taken together, we highlighted a NB-associated SNP rs3813832 T>C in Chinese population and found that *YTHDC1* acts as a NB oncogene.

RNA m^6^A methylation is an important epigenetic transcriptional modification and has the highest abundance in eukaryotes [24]. Its methylation balance depends on the synergy between the methyltransferase complex (eraser) and the demethylase (writer), and its function depends on the m^6^A binding protein (reader) [38]. Under their dynamic regulation, m^6^A plays an important regulatory role in various physiological processes [39]. Multiple evidence suggested that abnormalities of m^6^A modification are associated with human carcinogenesis, including hepatoblastoma [40], Wilms tumor [41], bladder cancer [34], and endometrial cancer [42]. The genetic variants in critical m^6^A modification genes may affect the individual’s susceptibility risk to cancers. Zeng et al. [43] genotyped 6 SNPs in *FTO* gene in samples of 537 breast cancer cases and 537 controls. Of the 6 SNPs analyzed, rs16953002 AA genotype significantly predisposed to a higher breast cancer risk compared to GG genotype, whereas rs1477196 AA genotype was associated with decreased breast cancer risk compared to GG genotype. In addition, Meng et al. [30] demonstrated that *SND1* gene rs118049207 could significantly increase the risk of colorectal cancer in the Nanjing and Beijing population of China. Further research revealed the rs118049207 acts as an enhancer of the *SND1* intron driven by *DMRT3*. Compared with adjacent normal tissues, the expression of *SND1* mRNA in colorectal tumor tissues increased significantly. They speculated that the genetic variation of m^6^A-modified genes might be a promising predictor of colorectal cancer risk. Polymorphisms in *METTL14* gene [44] and *METTL3* gene [45] (m^6^A methyltransferases) were also reported to be associated with predisposition to neuroblastoma. More recently, our group found that *YTHDC1* rs2293596 T>C polymorphism predisposed to hepatoblastoma and *YTHDC1* gene polymorphisms may have a cumulative effect on hepatoblastoma risk [46]. We also found that *YTHDC1* rs2293595 was associated with a significant inverse association with risk of glioma [33]. The combined effect of *YTHDC1* polymorphisms significantly increases Wilms tumor susceptibility [47]. These results indicated that m^6^A gene variants played an extremely important role in the development of tumors.

The m^6^A binding proteins (YTHDF1-3, YTHDC1, and YTHDC2) that belong to the YTH domain family are highly conserved in eukaryotic cells [48]. YTHDC1, as an important m^6^A recognition protein, is involved in the regulation of mRNA shear and mRNA nucleus [26, 49]. Although the function of YTHDC1 is still not fully understood, studies have shown that it plays a regulatory role in pre-mRNA splicing by interacting with SR protein. Based on the combination of transcriptome and PAR-CLIP sequencing technology, Xiao et al. [49] circumstantiated that m^6^A recruited the precursor mRNA splicing factor SRSF3 through its binding protein YTHDC1 to promote its binding to mRNA, while inhibiting the splicing factor SRSF10 binding to mRNA, thereby it promoted that m^6^A retains modified exons. In addition, some studies also reported that that YTHDC1 interacted with other splicing factors and could be used as potential tumor suppressors for endometrial cancer [42]. The expression of YTHDC1 has been detected in a panel of prostate cell lines and not in the benign prostate cell lines, indicating that YTHDC1 may function as an oncogene in prostate cancer [50]. These findings indicated that YTHDC1 might play an important role in the process of tumor development.

So far, the association between *YTHDC1* gene polymorphisms and NB susceptibility is unclear. Therefore, we explore the correlation between *YTHDC1* gene polymorphisms and NB susceptibility based on an eight-center case-control study. Our results showed that rs3813832 T>C could significantly reduce the risk of NB. In addition, stratified analysis revealed that individuals with 3 protective genotypes contributed to a decreased risk of NB in certain subgroups. Subsequently, we further explored whether the expression of YTHDC1 affected the occurrence and development of NB through cell experiments. Based on CCK-8, cell scratch, and cell migration experiments, we found that silencing *YTHDC1* would significantly inhibit the proliferation and migration of SK-N-BE (2) and SK-N-SH cells. Among three pairs of siRNA series, the inhibitory ability of siRNA-554 was the strongest, followed by siRNA-702, while the inhibitory ability of siRNA-488 was relatively weak. Combining these siRNA target sequences and the comprehensive analysis of rs3813832 T>C polymorphism site, we found that the rs3813832 T>C site was located in the coding sequence region of *YTHDC1*, and it was just located in the downstream region near siRNA-554. This finding indicated that the rs3813832 T>C polymorphic site might reduce the expression of *YTHDC1* by changing the amino acid composition of YTHDC1, and decrease the risk of individuals suffering from NB. These amino composition changes might affect the pre-mRNA splicing and transport of downstream RNA molecules, and thus ultimately inhibit the proliferation and metastasis of NB. The exact molecular mechanism of *YTHDC1* rs3813832 T>C on NB risk is worth further exploring.

Although this study has made some important discoveries, there are still several limitations. First of all, the subjects of this study were all Han population and could not represent the overall level of the Chinese population. In addition, the number of subjects was not large enough, which limited the statistical ability to some extent. It is necessary to further increase the number of research samples in future research. Finally, the research on YTHDC1 and NB proliferation/metastasis-related molecular mechanisms needs more exploration. Of note, the potential mechanism of how these SNPs impacts NB proliferation/metastasis awaits to be elucidated.

In conclusion, our findings indicated that *YTHDC1* rs3813832 T>C could significantly reduce the susceptibility of NB. In addition, individuals with multiple protective genotypes were less likely to suffer from NB. Silencing *YTHDC1* would inhibit the proliferation and migration of NB. The specific role and regulation mechanism of *YTHDC1* rs3813832 T>C in the occurrence and development of NB still needs to be fully elucidated.

## MATERIALS AND METHODS

### Study population

A total of 898 patients with NB and 1734 controls were recruited from eight hospitals in Guangzhou, Zhengzhou, Changsha, Wenzhou, Taiyuan, Xi’an, Kunming, and Shenyang (Supplementary Table 1). All cases were newly diagnosed and confirmed as NB by pathology. Participants without NB were recruited as healthy controls during the same period in the same geographic area. Specific recruitment details and selection criteria have been described in our previous studies [44, 51]. Informed consent forms were obtained from the parents or guardians of all participants. Our study has been approved by the Institutional Review Board of Guangzhou Women and Children’s Medical Center (Ethic approve No: 201929300).

### Genotyping

The criteria for choosing potential functional polymorphisms of the *YTHDC1* gene have been reported minutely in our previous study [46]. The selection criteria were as follows: SNPs are located in the in the 5′- flanking region, exon, 5′- untranslated region (5′UTR), and 3′UTR of *YTHDC1*; SNPs should be functional variations predicted by SNPinfo (https://snpinfo.niehs.nih.gov/snpinfo/snpfunc.html); the minor allele frequencies should be >5% in Chinese Han population; no significant linkage disequilibrium (LD) existed between selected SNPs (R^2^ < 0.8). We first screened out 31 potentially functional SNPs in *YTHDC1* via SNPinfo [46]. Among them, rs2293596 T>C and rs223595 T>C are located in the 3′UTR, predicting that they are related to microRNA binding. The rs3813832 T>C polymorphism was predicted to contribute to amino acid alteration. LDlink software (https://ldlink.nci.nih.gov/) result indicated that there was no significant LD (R^2^ < 0.8) among these three selected SNPs (R^2^ = 0.153 between rs2293596 and rs2293595; R^2^ = 0.082 between rs2293596 and rs3813832; R^2^ = 0.537 between rs2293595 and rs3813832). Genomic DNA was extracted from the participants’ venous blood samples. DNA samples were genotyped for the *YTHDC1* SNPs (rs2293596 T>C, rs2293595 T>C, and rs3813832 T>C) by ABI 7900HT real-time quantitative PCR instrument (Applied Biosystem, USA). In addition, 10% of DNA samples were chosen for repeated genotyping and the genotyping accuracy rate was 100%.

### Cell culture

Human neuroblastoma cell lines SK-N-BE (2) and SK-N-SH were purchased from the American Type Culture Collection (ATCC, USA). All cells were cultured in EMEM medium (ATCC, USA) containing 10% of fetal bovine serum (FBS; Gibco, USA), in an incubator at 37°C with 95% humidity and 5% CO_2_.

### Cell transfection

Three siRNAs (siRNA-488, siRNA-554, and siRNA-702) targeting *YTHDC1* gene were designed and synthesized by OBIO Biotechnology Co., Ltd (Shanghai, China). A scrambled siRNA (siRNA-NC) was synthesized as a negative control. All siRNAs sequences were listed in Supplementary Table 2. SK-N-BE (2) and SK-N-SH cells (4 × 10^5^ cells/well) were added to 6-well plates for 24 h in EMEM with 10% FBS before transfection. SK-N-BE (2) and SK-N-SH cells were transfected with *YTHDC1* siRNAs in Lipofectamine^™^ RNAiMAX Transfection Reagent (Thermo Fisher, USA). After transfection, cells were cultured in the incubator for 24 h for subsequent experiments.

### Cell proliferation assay

The CCK-8 kit (Dojindo Molecular Technologies, Japan) was used to measure the cell viability. SK-N-BE (2) and SK-N-SH cells (4 × 10^3^ cells/well) were transferred to 96-well plates and then transfected by *YTHDC1* siRNAs after 24 h. The optical density values at 450 nm (OD450) of SK-N-BE (2) and SK-N-SH cells treatment with CCK-8 reagent after transfection for 24 h, 48 h, and 72 h were detected by a microplate reader (Thermo Fisher, USA).

### Western blotting

The specific steps of Western blotting have been described in a previously published article [52]. After the incubation with anti-YTHDC1 antibody (1:1000), anti-β-actin antibody (1:1000), the membranes were incubated with peroxidase (HRP)-conjugated secondary antibody (1:4000). All the antibodies employed in this study were listed in Supplementary Table 3. The protein bands were detected using SuperSignal^™^ West Femto maximum sensitivity substrate (Thermo Fisher, USA) and a ChemiDOCTM XRS+ imaging system (Bio-rad, USA). The signal intensities of different protein bands were analyzed with ImageJ software (NIH, USA).

### Wound healing assay

SK-N-BE (2) and SK-N-SH cells (2.0 × 10^5^ cells/well) were transferred into 6-well plates and incubated for 24 h and then transfected with *YTHDC1* siRNAs. The cell scratches were performed with 10 μL pipette tips, and the cells were cultured with Opti-MEM (Gibco, USA) in the incubator. After incubation for 48 h, the cells were imaged with the fluorescence inverted microscope (Leica, Germany). The cell migration distances were calculated by Image-Pro Plus 6.0 software (Media Cybernetics, USA).

### Migration assay

The migration ability of SK-N-BE (2) and SK-N-SH cells transfected with siRNAs were evaluated using transwell assay in the 24-well plates. These cells transfected with siRNAs (2.5 × 10^4^ cells) were seeded into the 8 μm transwell upper chamber (BD Biosciences, USA) in 150 μL of EMEM basic medium. EMEM medium (700 μL) with 10% FBS was used as a chemo-attractant in the lower chamber. After 24 h, the cells that migrated to the lower surface of chamber were fixed with 70% absolute ethanol and stained with 0.1% crystal violet and imaged using the inverted microscope (Leica, Germany).

### Statistical analysis

In this study, statistical analysis was performed by SAS 9.4 software (SAS Institute Inc, USA). The distribution of sample characteristics between all NB cases and the control group was verified using a two-sided *χ*^2^ test. The goodness-of-fit chi-square test was used to test whether the genotype frequency in the control conforms to HWE. Based on unconditional logistic regression analysis, the AOR and 95% CI were calculated to assess the association between *YTHDC1* polymorphisms and NB risk. In addition, the cell functional experiments were performed independently for three times and the relevant data obtained were displayed with the mean ± SD. The SPSS 20.0 software (IBM, USA) was used for statistical analysis based on the least difference test by one-way analysis of variance (ANOVA). The standard with statistical significance were ^*^P value < 0.05 and ^**^P value < 0.01.

## Supplementary Materials

Supplementary Tables
