# Supplementary material for: YTHDC1 gene polymorphisms and neuroblastoma susceptibility in Chinese children
Source: Aging (Albany NY). 2021 Dec 12;13(23):25426–39. doi: 10.18632/aging.203760 (PMC8714171; doi:10.18632/aging.203760)
Supplement: Supplementary Tables [file aging-13-203760-s001.pdf]

## SUPPLEMENTARY TABLES

**Supplementary Table 1. Frequency distribution of selected characteristics in neuroblastoma cases and cancer-free controls.**

| Variables | Combined subjects (8 Centers) | | | | |
|---|---|---|---|---|---|
| | Cases (*n* = 898) | | Controls (*n* = 1734) | | $P^a$ |
| | No. | % | No. | % | |
| Age range, month | 0.00–176.00 | | 0.004–156.00 | | 0.155 |
| Mean ± SD | 33.11 ± 28.07 | | 30.41 ± 24.90 | | |
| ≤18 | 344 | 38.31 | 714 | 41.18 | |
| >18 | 554 | 61.69 | 1020 | 58.82 | |
| Gender | | | | | 0.236 |
| Female | 407 | 45.32 | 744 | 42.91 | |
| Male | 491 | 54.68 | 990 | 57.09 | |
| INSS stages | | | | | |
| I | 310 | 34.52 | / | / | |
| II | 160 | 17.82 | / | / | |
| III | 163 | 18.15 | / | / | |
| IV | 231 | 25.72 | / | / | |
| 4s | 18 | 2.00 | / | / | |
| NA | 16 | 1.78 | / | / | |
| Sites of origin | | | | | |
| Adrenal gland | 248 | 27.62 | / | / | |
| Retroperitoneal region | 319 | 35.52 | / | / | |
| Mediastinum | 214 | 23.83 | / | / | |
| Other region | 105 | 11.69 | / | / | |
| NA | 12 | 1.34 | / | / | |

Abbreviation: SD: standard deviation. [a]Two-sided $\chi^2$ test for distributions between neuroblastoma cases and cancer-free controls.

**Supplementary Table 2. Target sites and sequences of *YTHDC1* siRNAs.**

| siRNA names | Sequences (5′-3′) |
|---|---|
| YTHDC1 siRNA-488 | CGAAUGGAAUCUACUGAUATT<br>UAUCAGUAGAUUCCAUUCGTT |
| YTHDC1 siRNA-554 | CCACUGAGCUCAUCUGUUATT<br>UAACAGAUGAGCUCAGUGGTT |
| YTHDC1 siRNA-702 | CCAGAGAACCUUAUAAGAATT<br>UUCUUAUAAGGUUCUCUGGTT |
| YTHDC1 siRNA-NC | UUCUCCGAACGUGUCACGUTT<br>ACGUGACACGUUCGGAGAATT |

Abbreviation: NC: scrambled control.

**Supplementary Table 3. Antibodies used.**

| Antibodies | Company | Item number |
|---|---|---|
| anti-YTHDC1 | Abcam | Cat#ab122340 |
| anti-β-actin | Cell Signaling Technology | Cat#4970S |
| HRP-conjugated goat anti-rabbit IgG | Cell Signaling Technology | Cat#7074P2 |
